# N-Glycosylation of the SARS-CoV-2 Receptor Binding Domain Is Important for Functional Expression in Plants

**DOI:** 10.3389/fpls.2021.689104

**Published:** 2021-06-15

**Authors:** Yun-Ji Shin, Julia König-Beihammer, Ulrike Vavra, Jennifer Schwestka, Nikolaus F. Kienzl, Miriam Klausberger, Elisabeth Laurent, Clemens Grünwald-Gruber, Klemens Vierlinger, Manuela Hofner, Emmanuel Margolin, Andreas Weinhäusel, Eva Stöger, Lukas Mach, Richard Strasser

**Affiliations:** ^1^Department of Applied Genetics and Cell Biology, Institute of Plant Biotechnology and Cell Biology, University of Natural Resources and Life Sciences, Vienna, Vienna, Austria; ^2^Department of Biotechnology, University of Natural Resources and Life Sciences, Vienna, Vienna, Austria; ^3^Department of Biotechnology, Core Facility Biomolecular and Cellular Analysis, University of Natural Resources and Life Sciences, Vienna, Vienna, Austria; ^4^Department of Chemistry, Core Facility Mass Spectrometry, University of Natural Resources and Life Sciences, Vienna, Vienna, Austria; ^5^Competence Unit Molecular Diagnostics, Center for Health and Bioresources, AIT Austrian Institute of Technology GmbH, Vienna, Austria; ^6^Division of Medical Virology, Department of Pathology, Faculty of Health Sciences, University of Cape Town, Cape Town, South Africa; ^7^Biopharming Research Unit, Department of Molecular and Cell Biology, University of Cape Town, Cape Town, South Africa

**Keywords:** COVID-19, glycoprotein, glycosylation, posttranslational modification, SARS-CoV-2, virus

## Abstract

*Nicotiana benthamiana* is used worldwide as production host for recombinant proteins. Many recombinant proteins such as monoclonal antibodies, growth factors or viral antigens require posttranslational modifications like glycosylation for their function. Here, we transiently expressed different variants of the glycosylated receptor binding domain (RBD) from the SARS-CoV-2 spike protein in *N. benthamiana*. We characterized the impact of variations in RBD-length and posttranslational modifications on protein expression, yield and functionality. We found that a truncated RBD variant (RBD-215) consisting of amino acids Arg319-Leu533 can be efficiently expressed as a secreted soluble protein. Purified RBD-215 was mainly present as a monomer and showed binding to the conformation-dependent antibody CR3022, the cellular receptor angiotensin converting enzyme 2 (ACE2) and to antibodies present in convalescent sera. Expression of RBD-215 in glycoengineered ΔXT/FT plants resulted in the generation of complex N-glycans on both N-glycosylation sites. While site-directed mutagenesis showed that the N-glycans are important for proper RBD folding, differences in N-glycan processing had no effect on protein expression and function.

## Introduction

The ongoing COVID-19 pandemic underscores the urgency to increase the preparedness for future virus outbreaks and to establish countermeasures such as platform technologies to produce recombinant proteins for subunit vaccines or viral antigens for diagnostic tests (Amanat et al., 2020). High-level expression of authentic proteins is essential for the economic production of viral glycoproteins for different applications (Rybicki, 2009; Schillberg et al., 2019). To achieve high-level expression of recombinant proteins in plants, promoters or replicons derived from plant viruses are frequently used (Marillonnet et al., 2005; Sainsbury et al., 2009) and combined with synthetic gene approaches, such as codon optimization and signal peptide selection (Webster et al., 2017; Margolin et al., 2018). Despite these efforts, recombinant viral glycoproteins often accumulate at low levels in plants (Margolin et al., 2018). They are poorly processed (Le Mauff et al., 2015) and their expression can be associated with a stress response in the host leading to massive tissue necrosis (Phoolcharoen et al., 2011; Diego-Martin et al., 2020). The expression of the HIV-1 Env glycoprotein in *N. benthamiana* leaves, for instance, resulted in a severe phenotype and activation of the unfolded protein response (UPR), suggesting that the host biosynthetic machinery does not support efficient production of the protein (Margolin et al., 2019, 2020a).

Viral envelope or spike proteins that are used as subunit vaccines or in serological assays to detect neutralizing antibodies are often heavily glycosylated. The SARS-CoV-2 spike trimer contains 66 N-glycosylation sites that are highly occupied with N-glycans (Watanabe et al., 2020). The major folding pathway for glycoproteins in the ER involves the lectin chaperone calreticulin (CRT) and its membrane bound homolog calnexin (CNX) which bind to monoglucosylated N-glycans on substrate proteins and promote their folding (Kozlov and Gehring, 2020). CRT and CNX have been found associated with the HIV-1 Env glycoprotein and have an impact on folding of Env in mammalian cells (Otteken and Moss, 1996; Papandréou et al., 2010). Similarly, it was shown that binding of the SARS-CoV spike protein to CNX is critical for SARS-CoV infection (Fukushi et al., 2012). CNX promoted the folding of glycosylated spike protein during virus production and the progeny acquired infectious ability. CRT, on the other hand, did not associate with the SARS-CoV spike protein revealing different requirements of viral glycoproteins for lectin chaperone-mediated protein folding. In *N. benthamiana*, overexpression of human CRT increased the overall yield of several recombinant viral glycoproteins, including HIV-1 Env (Margolin et al., 2020a) and the ectodomain of the SARS-CoV-2 spike protein (Margolin et al., 2020c) and attenuated ER stress responses associated with viral glycoprotein expression. These findings underscore the importance of N-glycosylation and N-glycan-dependent quality control processes for recombinant protein production and reveal limitations that have to be addressed to make plant-based expression platforms such as *N. benthamiana* more attractive for economic production (Dicker and Strasser, 2015).

Here, we investigated the role of N-glycosylation for expression and function of the receptor binding domain (RBD) from the SARS-CoV-2 spike protein. Recombinant RBD can be used for vaccination approaches (Yang et al., 2020) or for serological assays to determine the presence and quality of an immune response against SARS-CoV-2 (Stadlbauer et al., 2020; Klausberger et al., 2021). The SARS-CoV-2 RBD has two N-glycosylation sites (N331 and N343) that are fully glycosylated when expressed in heterologous expression systems (Antonopoulos et al., 2020; Shajahan et al., 2020; Watanabe et al., 2020). Our data show that N-glycans on the RBD from the SARS-CoV-2 spike protein are important for protein folding and efficient RBD production as functional protein.

## Results

## RBD Is Present as a Homodimer and Non-functional After Purification From *N. benthamiana*

**FIGURE 1:**
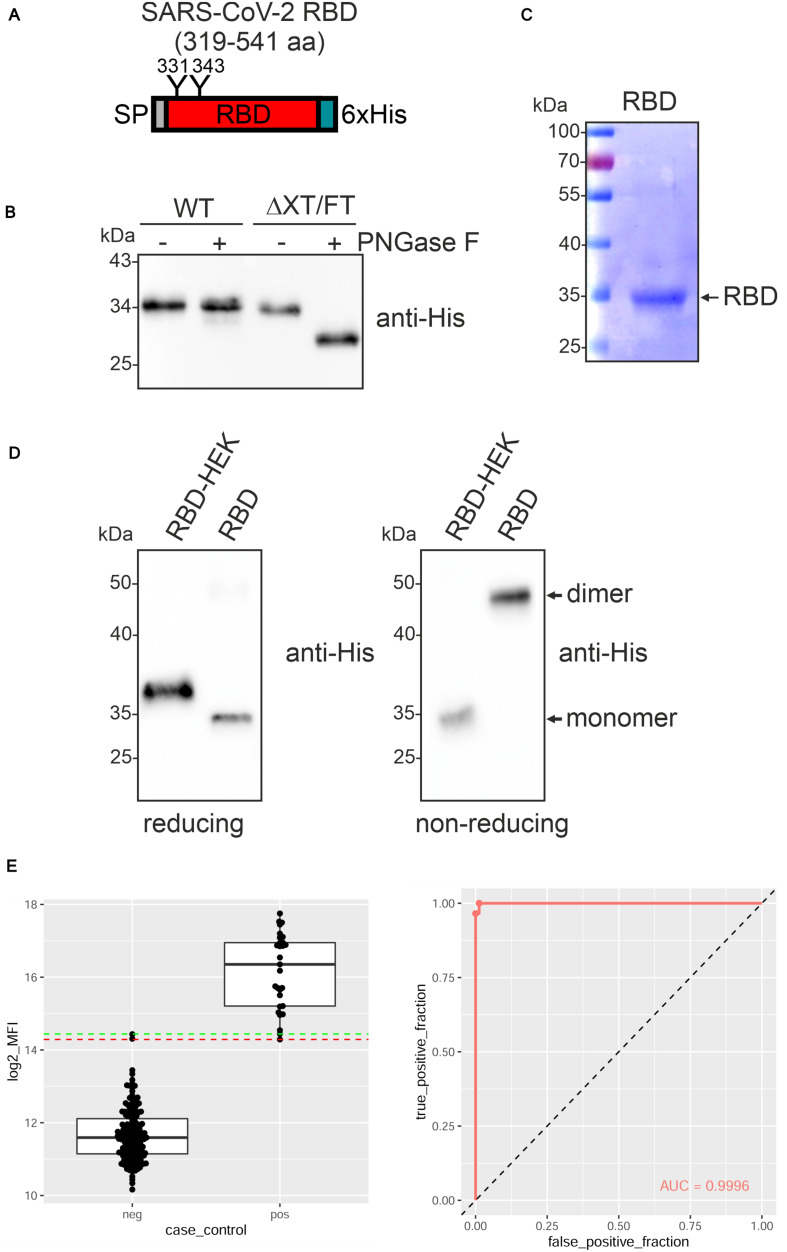
SARS-CoV-2 RBD is poorly expressed in *Nicotiana benthamiana*. **(A)** Schematic illustration of the SARS-CoV-2 RBD variant that was expressed. The position of the signal peptide (SP), the two N-glycans at position N331 and N343 and the C-terminal 6x histidine tag are indicated. **(B)** Protein extracts from infiltrated *N. benthamiana* wild-type (WT) or ΔXT/FT plants were subjected to PNGase F digestion and analyzed by immunoblots with antibodies against the 6x histidine-tag. **(C)** RBD produced in ΔXT/FT was purified from the apoplastic fluid 4 days after infiltration, subjected to SDS-PAGE under reducing conditions and stained with Coomassie Brilliant Blue. **(D)** Immunoblot analysis of RBD produced in HEK293 cells (RBD-HEK, 319–541 aa) and RBD produced in *N. benthamiana*. SDS-PAGE was carried out under reducing and non-reducing conditions. **(E)** Binding of sera from blood donors collected prior to 2018 (neg, *n* = 163) and sera from SARS-CoV-2 exposed individuals (pos, *n* = 26) to plant-produced RBD. Binding was analyzed using a Luminex bead-based assay and the median fluorescent intensity (log2 MFI) is shown. The lines indicating the 100% sensitivity cut-off (red) and the 100% specificity cut-off (green) as well as the receiver operating characteristic (ROC) curve and the area under the curve (AUC) are shown.

The receptor-binding domain (RBD) of the SARS-CoV-2 spike protein (amino acids R319-F541) (Figure 1A and Supplementary Figure 1) (Amanat et al., 2020; Lan et al., 2020) fused to the barley α-amylase signal peptide and a C-terminal polyhistidine tag was transiently expressed in leaves of *N. benthamiana* wild-type and glycoengineered ΔXT/FT plants (Strasser et al., 2008). A band of approximately 34 kDa was detectable and the expression levels were comparable in both *N. benthamiana* lines (Figure 1B). While recombinant RBD expressed in ΔXT/FT was sensitive to PNGase F digestion, RBD from wild-type was fully resistant indicating the presence of complex N-glycans with core α1,3-fucose that prevents cleavage by PNGase F in the wild-type derived RBD. For further characterization, RBD was purified from the apoplastic fluid of ΔXT/FT by immobilized metal ion affinity chromatography (IMAC) (Figure 1C). During purification, we noticed that the protein was less stable and prone to aggregate. Consequently, the overall yield varied from purification to purification and ranged from 1 to 10 μg/g fresh leaf. Purified plant-derived RBD migrated faster on immunoblots compared to RBD produced in HEK293 cells (RBD-HEK) which is presumably caused by divergent N-glycan processing (Figure 1D). SDS-PAGE under non-reducing conditions followed by immunoblotting revealed a major band of approximately 50 kDa for plant-produced RBD, indicating the presence of dimers. Homodimer formation for plant and mammalian-cell produced RBD was recently confirmed by size-exclusion chromatography and is likely promoted by incorrectly formed disulfide bonds (Klausberger et al., 2021).

To examine whether plant-produced RBD is correctly folded, we analyzed the binding to human angiotensin converting enzyme 2 (ACE2) by ELISA. While RBD-HEK displayed the expected interaction with an ACE2-Fc fusion protein (see below), binding by plant-produced RBD was not consistently observed (data not shown). Similar results were obtained when the monoclonal antibody CR3022 was used as a capture antibody. CR3022 binds to a conformational RBD epitope (Yuan et al., 2020) and impaired binding to CR3022 is indicative of improper folding of the plant-produced RBD. Despite the failure to bind ACE2-Fc and CR3022, plant-produced RBD reacted with convalescent sera indicating the presence of epitopes that are recognized by polyclonal antibodies present in SARS-CoV-2 exposed individuals (Figure 1E).

### RBD-KDEL Is Unstable When Transiently Expressed in *N. benthamiana*

**FIGURE 2:**
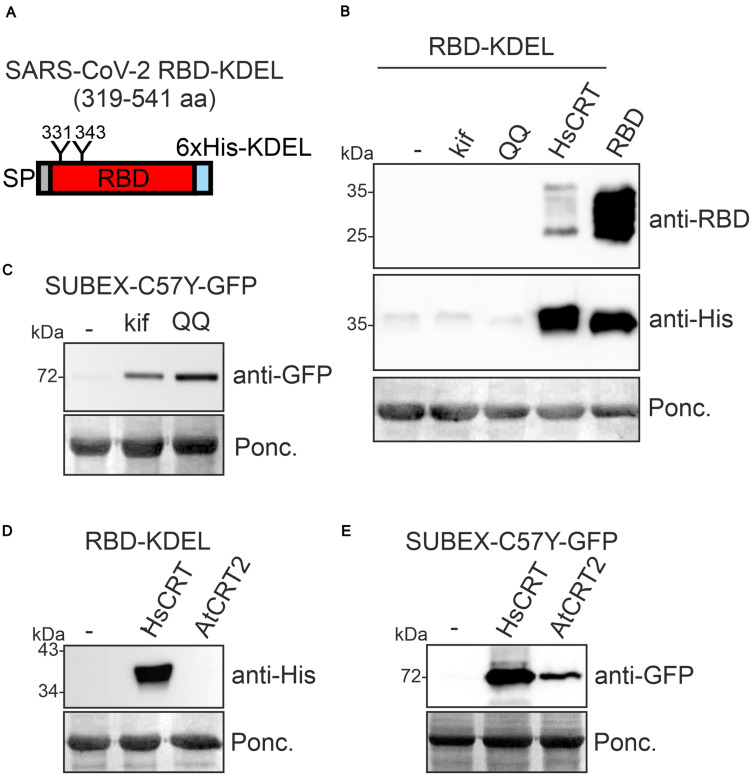
Co-expression of human CRT results in RBD-KDEL accumulation in *Nicotiana benthamiana*. **(A)** Schematic illustration of the expressed SARS-CoV-2 RBD-KDEL variant. **(B)** RBD-KDEL was co-expressed with 50 μM kifunensine (kif), *Arabidopsis* CDC48A-QQ (QQ) or human CRT (HsCRT). Samples were analyzed by immunoblotting 3 days after infiltration of *N. benthamiana* WT. Ponceau S staining (Ponc.) is shown as a loading control. RBD expression was included for comparison. **(C)** SUBEX-C57Y-GFP was co-expressed with kif or *Arabidopsis* CDC48A-QQ (QQ) and analyzed 3 days after infiltration of *N. benthamiana* WT. **(D)** RBD-KDEL was co-expressed with HsCRT or *Arabidopsis* CRT2 (AtCRT2). Both CRT variants were expressed with the pEAQ-*HT* vector and expression was analyzed 4 days after infiltration of *N. benthamiana* WT. **(E)** SUBEX-C57Y-GFP was co-expressed with HsCRT or AtCRT2 and analyzed 3 days after infiltration of *N. benthamiana* WT.

For several recombinant proteins the attachment of a HDEL/KDEL Golgi-to-ER retrieval signal resulted in ER accumulation and improved expression levels in plants (Petruccelli et al., 2006; Loos et al., 2011). To see if increased ER-retention is beneficial for RBD production, we expressed RBD-KDEL transiently in *N. benthamiana* leaves (Figure 2A). Surprisingly, RBD-KDEL, which differs from RBD only by the presence of the KDEL tetrapeptide inserted after the polyhistidine tag (Supplementary Figure 1), was not detectable in crude protein extracts from infiltrated leaves (Figure 2B, lane 1). Incorrect disulfide bond formation together with prolonged ER retention could target RBD-KDEL to clearance by ER-associated degradation (ERAD) which is the major pathway for the degradation of misfolded glycoproteins from the ER (Hüttner and Strasser, 2012; Shin et al., 2018). To examine whether ERAD plays a role for the degradation of RBD-KDEL, we co-infiltrated RBD-KDEL together with the ERAD inhibitor kifunensine, which blocks the α-mannosidases that generate the N-glycan degradation signal required for ERAD of glycoproteins (Hüttner et al., 2014b). In addition, we co-infiltrated a dominant-negative variant of the *Arabidopsis* AAA ATPase CDC48A (CDC48A-QQ) which was previously shown to block ERAD (Müller et al., 2005). While kifunensine and CDC48A-QQ blocked the degradation of the glycosylated ERAD substrate SUBEX-C57Y-GFP (Hüttner et al., 2014a; Figure 2C), there was no improvement in the accumulation of RBD-KDEL indicating that the lack of protein was not due to glycan-dependent ERAD (Figure 2B). When we co-expressed human CRT, on the other hand, we could clearly detect RBD-KDEL on immunoblots (Figures 2B,D). By contrast, co-expression of *Arabidopsis* CRT2, which stabilized the ERAD substrate SUBEX-C57Y-GFP (Figure 2E), or co-expression of *Arabidopsis* CNX1, did not have an impact on RBD-KDEL (Figure 2D and Supplementary Figure 2). Taken together, these data indicate that RBD-KDEL is poorly expressed as a soluble protein in *N. benthamiana*.

### Human CRT Retains RBD in the ER

**FIGURE 3:**
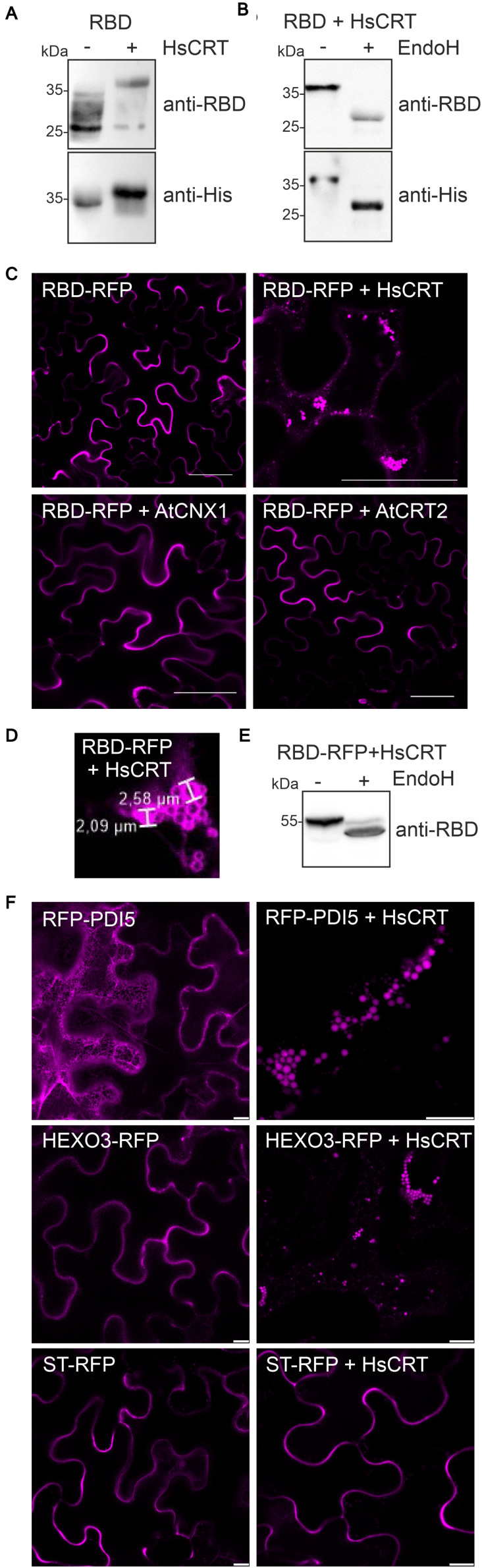
Human CRT retains glycoproteins in intracellular compartments. **(A)** HsCRT was co-expressed with RBD and analyzed 4 days after infiltration of *Nicotiana benthamiana* WT by immunoblotting with antibodies against RBD or the 6x histidine tag. **(B)** Endo H digestion of RBD co-expressed with. HsCRT.**(C)** Confocal microscopy of RBD-RFP co-expressed with HsCRT, AtCNX1 or AtCRT2. Scale bars = 50 μm. **(D)** Enlarged confocal image of RBD-RFP co-expressed with HsCRT. **(E)** Endo H digestion and immunoblotting of RBD-RFP co-expressed with HsCRT. **(F)** RFP-PDI5 co-expressed with/without HsCRT, HEXO3-RFP co-expressed with/without HsCRT, ST-RFP co-expressed with/without HsCRT. Images were taken 3 days after infiltration. Scale bars = 10 μm.

Next, we examined the effect of human CRT on RBD expression. When we co-expressed human CRT, the RBD expression levels appeared unchanged, but the mobility in SDS-PAGE was altered indicating differences in N-glycan processing (Figure 3A). We hypothesized that binding of human CRT prevents the trimming of monoglucosylated N-glycans (Glc_1_Man_9_GlcNAc_2_) to complex ones and causes the observed slower migration. Upon Endo H digestion a shift in mobility was detectable showing that RBD co-expressed with human CRT harbors oligomannosidic N-glycans (Figure 3B). The additional RBD bands that are detectable with the anti-RBD antibody are likely the result of proteolytic processing at the C-terminus (Figure 3A) which might take place in the apoplast.

The previous experiments indicated that RBD is trafficking through the Golgi where oligomannosidic N-glycans are processed to complex ones (Figure 1B). Co-expression of human CRT prevents the maturation to complex N-glycans by retaining RBD in the ER or by protecting the N-glycans from processing in the Golgi. To examine the effect of CRT on the subcellular localization of RBD we replaced the polyhistidine-tag on RBD with the red fluorescent protein (RFP) and analyzed its localization in *N. benthamiana* leaf epidermal cells. Confocal microscopy confirmed that RBD-RFP was secreted to the apoplast (Figure 3C). By contrast, in the presence of human CRT, RBD-RFP was found in intracellular structures visually resembling protein bodies (Conley et al., 2009; Saberianfar et al., 2015) with a diameter of approximately 2.0–2.6 μm (Figures 3C,D). RBD-RFP expressed with human CRT displayed Endo H sensitive N-glycans, suggesting that the intracellular structures are ER-derived (Figure 3E). *Arabidopsis* CRT2 or CNX1 expression did not cause the localization in such cellular structures and RBD-RFP was still secreted to the apoplast (Figure 3C). To test if human CRT is specific for RBD or generally retaining glycoproteins in protein body-like structures, we co-expressed two glycoproteins, *Arabidopsis* RFP-PDI5 and *N. benthamiana* HEXO3-RFP (Farid et al., 2011; Shin et al., 2017), and the non-glycosylated ST-RFP fusion protein (Schoberer et al., 2019). While the two glycoproteins were found in protein body-like structures, ST-RFP was still detected in the apoplast (Figure 3F). This shows that human CRT specifically retains glycoproteins in ER-derived intracellular protein body-like structures but does not have a general effect on secretion of proteins.

### A Truncated RBD Variant Is Efficiently Produced in *N. benthamiana* and Properly Folded

The low expression levels of RBD and the tendency for homodimer formation is possibly caused by the presence of an unpaired cysteine residue (C538) at the C-terminus (Supplementary Figure 1) leading to intermolecular cross-linking, homodimer formation and aggregation. To improve the expression of the soluble monomeric form we expressed an RBD variant with the cysteine at position 538 substituted by an alanine residue transiently in *N. benthamiana*. While RBD-C538A could be detected on immunoblots with an RBD-specific polyclonal antibody, reduced signals were present on immunoblots probed with an anti-His antibody indicating that the C-terminus is either not accessible or unstable (Supplementary Figure 3). Moreover, SDS-PAGE separation under non-reducing conditions and immunoblot analysis with an RBD-specific antibody showed that considerable amounts of dimeric RBD-C538A are still present, despite the removal of the unpaired cysteine residue. This result indicates that other cysteines may also contribute to the formation of dimeric RBD variants.

**FIGURE 4:**
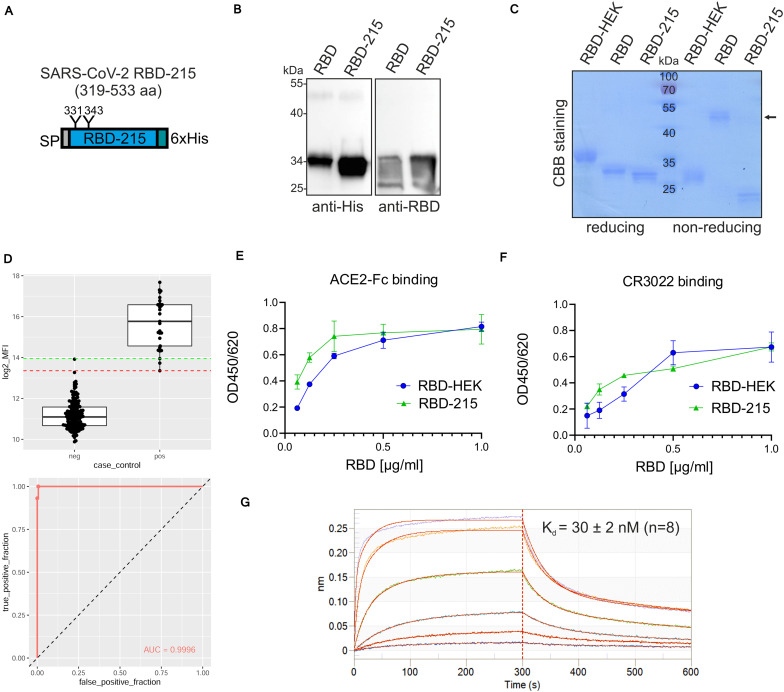
A plant-produced truncated RBD variant is functional. **(A)** Schematic illustration of the truncated RBD-215 variant. **(B)** Comparison of RBD and RBD-215 protein expression in leaf extracts of ΔXT/FT *Nicotiana benthamiana* analyzed by immunoblotting 4 days after infiltration. **(C)** RBD variants were purified from the apoplastic fluid 4 days after infiltration, analyzed by SDS-PAGE under reducing or non-reducing conditions, followed by Coomassie Brilliant Blue (CBB) staining. HEK293-produced RBD (RBD-HEK) was included for comparison. The altered mobility of RBD-HEK and the plant produced RBD variant is caused by differences in complex N-glycans. The arrow marks the position of the homodimer. **(D)** Binding of sera from healthy blood donors collected prior to 2018 (neg, *n* = 163) and sera from SARS-CoV-2 exposed individuals (pos, *n* = 26) to plant-produced RBD-215. Binding was analyzed using a Luminex bead-based assay and the median fluorescent intensity (log2 MFI) is shown. The lines indicating the 100% sensitivity cut-off (red) and the 100% specificity cut-off (green) as well as the receiver operating characteristic (ROC) curve and the area under the curve (AUC) are shown. **(E)** Binding of purified plant-produced RBD-215 and RBD-HEK to plates coated with ACE2-Fc or **(F)** antibody CR3022. Data are presented as mean ± SD (*n* = 3). **(G)** BLI analysis. Binding kinetics of the interaction between biotinylated mAb CR3022 loaded on SAX biosensors and RBD-215 at a concentration range of 1.2–300 nM. Representative real-time association and dissociation curves are shown.

Next, we expressed a truncated RBD variant (RBD-215: amino acids R319-L533) lacking the cysteine at position 538 (Figure 4A and Supplementary Figure 1). The expression level of RBD-215 was approximately two times higher than the RBD expression level (RBD-215: 117 ± 41 μg/g fresh leaves compared to 63 ± 10 μg/g for RBD, determined from four independent experiments) (Figure 4B). Consistent with the higher expression level, more of RBD-215 could be purified from the apoplastic fluid (10–20 μg/g fresh leaves). SDS-PAGE of IMAC-purified RBD-215 under non-reducing conditions showed primarily the monomeric form (Figure 4C) and size-exclusion chromatography revealed approximately 10% dimers (Supplementary Figure 4). MS analysis of peptides showed that both RBD-215 N-glycosylation sites contained exclusively Golgi-processed N-glycans (Supplementary Figure 5). GlcNAc_2_Man_3_GlcNAc_2_ (GnGn) was the major peak on both sites and low amounts of truncated N-glycans likely generated in the apoplast were present. While N343 was almost completely (>99%) occupied, site N331 displayed small amounts (<4%) of non-glycosylated peptide. A bead-based binding assay with sera from SARS-CoV-2 exposed individuals confirmed that recombinant RBD-215 is recognized as SARS-CoV-2 antigen with high sensitivity and specificity (Figure 4D). ELISA with ACE2-Fc and the conformation-dependent RBD antibody CR3022 showed that RBD-215 binds equally well like HEK293-produced RBD to ACE2-Fc and CR3022 (Figures 4E,F). Biolayer interferometry analysis revealed that RBD-215 has an affinity (K_*d*_ of 30 ± 2 nM, *n* = 8) for CR3022 (Figure 4G) that is comparable to recombinant RBD produced in mammalian or insect cells (Klausberger et al., 2021).

### N-Glycosylation Is Important for RBD-215 Production in *N. benthamiana*

**FIGURE 5:**
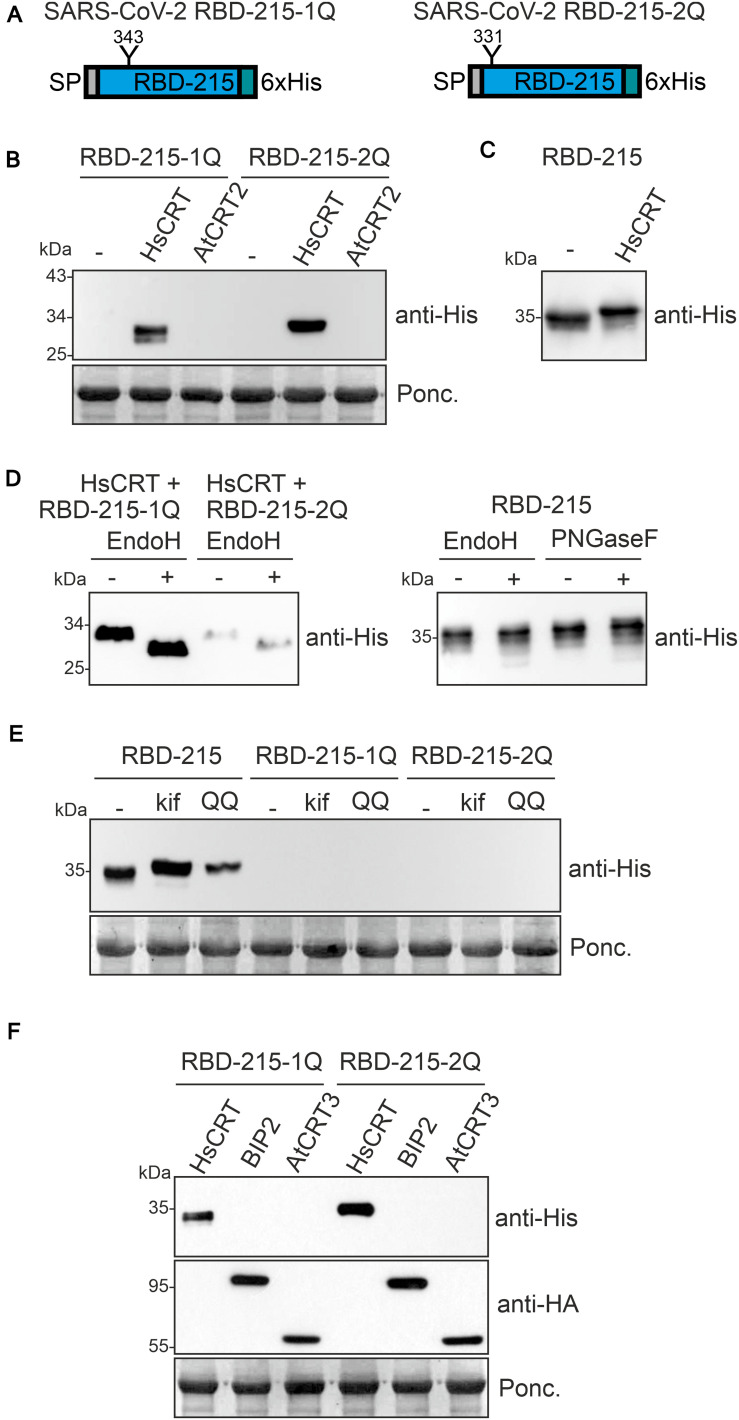
N-glycosylation at both sites of the truncated RBD variant is required for expression in *Nicotiana benthamiana*. **(A)** Schematic illustration of the mutated RBD-215 variants lacking the indicated N-glycosylation sites. **(B)** RBD-215-1Q and RBD-215-2Q were co-expressed with HsCRT or AtCRT2 and samples were analyzed 4 days after infiltration of *N. benthamiana* WT by immunoblotting. **(C)** RBD-215 co-expressed with HsCRT is shown as a control. **(D)** Protein extracts from infiltrated *N. benthamiana* WT were subjected to Endo H or PNGase F digestion and analyzed by immunoblotting with anti-His antibodies. **(E)** RBD-215-1Q and RBD-215-2Q were co-expressed with kif or *Arabidopsis* CDC48A-QQ (QQ). Samples were analyzed 3 days after infiltration of *N. benthamiana* WT. **(F)** RBD-215-1Q and RBD-215-2Q were co-expressed with *Arabidopsis* BIP2 or *Arabidopsis* CRT3 both carrying an HA-tag for detection.

The effect of the human lectin chaperone CRT on RBD variants suggests that N-glycosylation is important for ER-quality control and possibly also for protein folding. To characterize the effect of individual N-glycosylation site mutations on RBD-215 expression, we expressed the corresponding RBD-215 mutants in *N. benthamiana* leaves. In RBD-215-1Q, N331 was changed to Q331 and in RBD-215-2Q, N343 was changed to Q343 (Figure 5A). In crude protein extracts obtained 4 days after infiltration no protein was detectable on immunoblots. However, when co-expressed with human CRT, a specific band of expected size was found for both RBD-215 mutant variants (Figure 5B). By contrast, co-expression of *Arabidopsis* CRT2 from the same expression vector did neither result in the accumulation of RBD-215-1Q nor of RBD-215-2Q. Co-expression of human CRT resulted in reduced RBD-215 mobility on immunoblots indicating the presence of incompletely processed oligomannosidic N-glycans, but no overall increase in protein levels was detected (Figure 5C and Supplementary Figure 6). In contrast to RBD-215 that carries Endo H and PNGase F insensitive N-glycans when expressed in wild-type, both RBD-215 mutant variants were Endo H sensitive (Figure 5D). This finding is consistent with the CRT-mediated retention in ER-derived intracellular structures. Similar to RBD-KDEL, neither the treatment with the ERAD inhibitor kifunensine nor the expression of the dominant-negative ERAD factor CDC48A-QQ caused the accumulation of RBD-215-1Q or RBD-215-2Q (Figure 5E), suggesting that the RBD-215 mutant variants are not subjected to ERAD.

Our data show that *Arabidopsis* CRT2 is apparently less effective than human CRT in promoting accumulation of structurally compromised RBD variants in plants. To analyze the function of other members of the plant CRT family we co-expressed *Arabidopsis* CRT3. This CRT is specific for plants and has been found to interact with structurally defective *Arabidopsis* EF-Tu receptor (EFR) or BRASSINOSTEROID INSENSITIVE1 (BRI1) (Jin et al., 2009; Li et al., 2009; Saijo et al., 2009). Co-expression of RBD-215-1Q or RBD-215-2Q with HA-tagged *Arabidopsis* CRT3 did not have a comparable effect on the mutant variants as observed for human CRT expression (Figure 5F). Similarly, co-expression of the HSP70 family protein BIP2 that is involved in ER-retention and folding of defective proteins (Hong et al., 2008) did not improve RBD-215-1Q or RBD-215-2Q expression. To examine whether the effect of human CRT expression is N-glycan-dependent, we generated an RBD-215 variant (RBD-215-12Q) lacking both N-glycosylation sites. When infiltrated, no expression was detected on immunoblots and human CRT did not cause RBD-215-12Q accumulation (Supplementary Figure 7).

**FIGURE 6:**
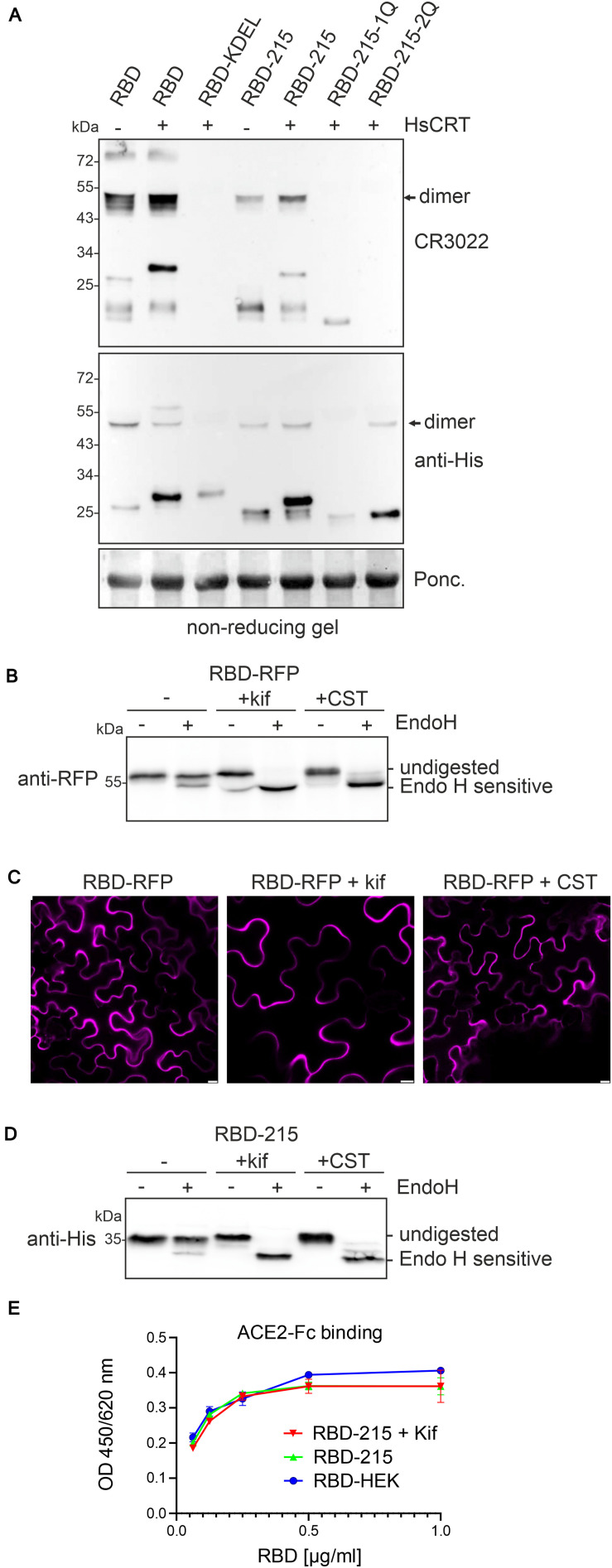
Human CRT has only a minor effect on folding of aberrant RBD variants. **(A)** The indicated RBD variants were expressed with or without HsCRT, crude protein extracts were subjected to SDS-PAGE under non-reducing conditions and analyzed by immunoblotting with anti-His or CR3022 antibody that binds to a conformational epitope on RBD. **(B)** Endo H digestion and immunoblot analysis of RBD-RFP (2 days after infiltration). **(C)** Confocal microscopy of RBD-RFP expressed in the presence of 50 μM kifunensine (kif) or 200 μM castanospermine (CST). Images were taken 2 days after infiltration. Scale bars = 10 μm. **(D)** Endo H digestion and immunoblot analysis of RBD-215 (3 days after infiltration) co-infiltrated with kifunensine. **(E)** ELISA of purified RBD-215 carrying oligomannosidic N-glycans. ACE2-Fc was coated and binding of RBD-215, RBD-215 + kif and RBD produced in HEK293 cells (RBD-HEK) was monitored with an anti-His antibody. Data are presented as mean ± SD (*n* = 3).

Expression of human CRT may suppress aggregate formation and promote folding of RBD-KDEL, RBD-215-1Q, and RBD-215-2Q leading to intracellular accumulation. To assess the folding of RBD variants we subjected crude protein extracts from leaves to SDS-PAGE under non-reducing conditions and used the conformation-dependent RBD-specific antibody CR3022 for detection on immunoblots (Figure 6A). RBD-215 and to some extent RBD and RBD-215-1Q reacted with CR3022. CR3022, on the other hand, did not bind to RBD-KDEL and RBD-215-2Q, suggesting that human CRT-mediated ER-retention does not substantially improve their folding.

Finally, we investigated the effect of the two N-glycan processing inhibitors kifunensine and castanospermine on the expression and subcellular localization of RBD-RFP. While kifunensine blocks the removal of α-mannose residues, castanospermine is an inhibitor of α-glucosidase I and II involved in the trimming of α-glucose residues from the transferred Glc_3_Man_9_GlcNAc_2_ N-glycan. When co-infiltrated into leaves, both pharmacological inhibitors rendered the protein fully susceptible to Endo H digestion which confirms their inhibitory effect on N-glycan processing. However, the inhibition of early steps in N-glycan processing did neither alter RBD-RFP protein expression nor block RBD-RFP secretion (Figures 6B,C). In agreement with these data, RBD-215 co-infiltrated with kifunensine or castanospermine was readily expressed and carried exclusively Endo H sensitive oligomannosidic N-glycans (Figure 6D). To confirm this finding, we expressed RBD-215 in the presence of kifunensine and purified the protein from the apoplastic fluid. RBD-215 + kif displayed mainly unprocessed Man_9_GlcNAc_2_ N-glycans (Supplementary Figure 8), showed comparable yields after purification from the apoplastic fluid and was functional (Figure 6E). In summary, our data strongly indicate that N-glycosylation is crucial for RBD folding when transiently produced in *N. benthamiana*, but the degree of N-glycan processing and type of attached N-glycans are not important for RBD production and secretion.

## Discussion

In this study, we analyzed the transient expression of different recombinant RBD variants in *N. benthamiana*. We expressed an RBD variant that was previously produced in mammalian or insect cells (Stadlbauer et al., 2020; Klausberger et al., 2021) and observed low expression levels and high amounts of homodimers. Recombinant SARS-CoV-2 RBD variants have recently been produced in *N. benthamiana* using different transient expression systems (Diego-Martin et al., 2020; Mamedov et al., 2020; Rattanapisit et al., 2020; Makatsa et al., 2021). Diego-Martin et al. (2020) used a MagnICON-based expression vector to produce a His-tagged RBD variant carrying the same amino acid region (R319-F541) as present in our RBD. In agreement with our data, low yields (2-4 μg/g fresh weight) and considerable amounts of higher molecular weight bands were reported. In the study from Rattanapisit et al. (2020), a longer RBD variant (amino acids F318-C617) was expressed. In this variant C538 can form a disulfide bridge with C590 (Walls et al., 2020) which may stabilize the protein conformation leading to a functional protein that binds to ACE2 and CR3022 (Rattanapisit et al., 2020). There is, however, another cysteine at position 617 that remains unpaired as it makes a disulfide bridge with C649 in the full-length spike protein (Walls et al., 2020). The overall yield of this longer RBD variant was in the same range (8 μg/g fresh weight) as observed for the other plant produced RBDs. Mamedov et al. (2020) reported the expression of an RBD variant consisting of amino acids R319-S591. Like in the RBD-215 variant, there is an even number of cysteine residues in the amino acid sequence and the yield after purification (10–20 μg/g fresh weight, using the pEAQ-*HT* expression vector) was comparable to our yield. Together with our results, these data show that the choice of the RBD amino acid region is crucial for obtaining functional soluble protein with low aggregate formation.

When we used a polyclonal antibody binding to the RBD from SARS-CoV-2, we frequently observed additional faster migrating bands for the different expressed RBD variants. These bands were not observed with the antibody against the C-terminal 6x His-tag, indicating that the C-terminus undergoes proteolysis. Co-expression of human CRT reduced these bands. This suggests that association with human CRT or retention in protein body-like structures prevents the cleavage which might occur in the apoplast. While the unwanted proteolytic processing has no effect on the homogeneity and quality of the IMAC-purified recombinant RBD variants, it contributes to the observed low yield.

Numerous and diverse mammalian proteins have been successfully expressed in the secretory pathway of plants indicating that the plant ER is generally well equipped for protein folding and co- or posttranslational modifications (Stoger et al., 2014; Margolin et al., 2020b). However, there are subtle differences in protein folding and modifications in the ER that cause, for example, underglycosylation of recombinant human proteins expressed in plants (Castilho et al., 2018) or affect the assembly of protein subunits. Co-expression of folding assistants, including the chaperone BIP or the human thioredoxin family protein ERp44 increased the overall yield of a recombinant dimeric IgA produced in *N. benthamiana* (Göritzer et al., 2020). Similarly, co-expression of human CRT improved the expression of viral glycoproteins like HIV gp140 (Margolin et al., 2020a) and the full-length SARS-CoV-2 spike ectodomain (Margolin et al., 2020c). In vitro assays have shown that mammalian CRT is very effective in suppressing the aggregation of glycosylated proteins (Stronge et al., 2001). This appears to apply also to human CRT expressed in plants. While we observed no major impact on the expression levels of RBD-215, RBD or RBD-RFP, human CRT increased the amounts of structurally compromised RBD variants like RBD-KDEL and the two RBD-215 N-glycosylation site mutants that may form insoluble aggregates. Upon human CRT expression, the proteins carried oligomannosidic N-glycans and were retained inside the cells in distinct cellular structures visually resembling ER-derived protein bodies. Although the basic function of plant and human CRTs is conserved, the overexpression of *Arabidopsis* CRT2 did not result in intracellular accumulation of RBD variants. The sequence identity between *Arabidopsis* CRT2 and human CRT is quite high in the globular N domain and the proline-rich P domain and amino acid residues involved in chaperone function are fully conserved (Christensen et al., 2008; Qiu et al., 2012a; Liu and Li, 2013). The C-terminal acidic domain that is important for calcium binding is less conserved, but both human CRT and *Arabidopsis* CRT2 harbor a similar negative net charge in this region (Qiu et al., 2012b). Further studies are needed to address the functional differences between human and plant CRTs. In mammalian cells, CNX interacts with the SARS-CoV spike protein and promotes its folding (Fukushi et al., 2012). While the role of CNX for SARS-CoV-2 spike protein folding is currently unknown, it is possible that co-expression of human CNX has also a positive effect on the accumulation of structurally compromised RBD.

Castanospermine and derivatives are considered as potential antiviral drugs and have been used to prevent SARS-CoV infections (Fukushi et al., 2012) and SARS-CoV-2 replication (Clarke et al., 2021). In the presence of castanospermine glucose trimming is blocked and no monoglucosylated N-glycans are formed. The unprocessed N-glycans cannot interact with the carbohydrate-binding site from CRT/CNX (Kozlov and Gehring, 2020) thus preventing entry of the CNX/CRT cycle. Our data with castanospermine provide hints that impaired glycan-dependent interaction with endogenous plant CRT or CNX does not severely affect RBD-RFP and RBD-215 folding. The major role of the RBD-215 N-glycans could therefore be a direct effect on folding of the protein.

A role of N-glycosylation in immune evasion by camouflaging immunogenic protein epitopes is well known for pathogenic viruses (Watanabe et al., 2020) and the presence or absence of N-glycans can have a profound effect on the virus infectivity, antigenicity and immunogenicity of recombinant antigens (Vigerust et al., 2007; Ringe et al., 2019; Li et al., 2020). N-glycans are important for the spike protein folding, for modulating the accessibility to host proteases, for shielding to avoid the detection by the immune response of infected individuals and for interaction with cellular receptors (Walls et al., 2016; Pallesen et al., 2017; Li et al., 2020; Pinto et al., 2020; Zhang et al., 2020). The two RBD N-glycans are not part of the receptor-binding motif and not directly involved in receptor binding (Walls et al., 2020; Yang et al., 2020). The N-glycosylation site at position N343 is conserved in sarbecoviruses (Watanabe et al., 2020). Mutations of the SARS-CoV-2 RBD N-glycosylation site N343 affected RBD expression in a yeast cell surface-display assay (Starr et al., 2020). By contrast, various mutations at site N331 did not have a major effect on protein expression. ACE2 binding affinity, on the other hand, was less altered in these N-glycosylation site mutants. In another recent study, RBD N-glycosylation site mutants were expressed in HEK293 cells without any obvious effects on expression levels, but decreased ACE2 binding upon removal of the N-glycans at both sites was reported (Azad et al., 2021). In agreement with the later finding, the glycan at position N343 may play an important role for the opening mechanism of the spike and stabilization of RBD in the up state that is required for ACE2 binding and cell entry (Sztain et al., 2021). In plants, neither the individual RBD-215 N-glycosylation mutants nor the RBD-215 variant lacking both N-glycosylation sites (RBD-215-12Q) could be produced as soluble proteins, suggesting that both N-glycans contribute to proper folding. The misfolded glycosylated variants are not subjected to glycan-dependent ERAD which is the canonical pathway for clearance of misfolded glycoproteins in plants, yeast and mammals (Hüttner and Strasser, 2012). This suggests that aberrant RBD variants either form insoluble aggregates or are subjected to clearance by alternative processes involving autophagy and vacuolar degradation. Alternatively, the possibility of degradation during secretion or in the apoplast cannot be completely excluded. In conclusion, our studies show that N-glycosylation is critical to produce functional recombinant RBD variants in plants. Encouragingly, this work also demonstrates the suitability of plants for the production of serology reagents, meriting further development of plant-based platforms as part of the response to future pandemic outbreaks.

## Materials and Methods

### RBD Cloning

The codon-optimized DNA sequence coding for the SARS-CoV-2 RBD (amino acids 319-541 from P0DTC2 – SPIKE_SARS2) from the first human isolate Wuhan-1 fused to the α-amylase signal peptide and a C-terminal hexahistidine tag was synthesized by GeneArt (Thermo Fisher Scientific). The DNA fragment was amplified by PCR with STRINGS-7F/STRINGS-8R (Supplementary Table 1), AgeI/XhoI digested and ligated into AgeI/XhoI digested plant expression vector pEAQ-*HT* (Sainsbury et al., 2009). To generate the pEAQ-RBD-KDEL expression vector, the sequence was amplified by PCR from the synthetic RBD DNA fragment with STRINGS-7F and RBD-6R and cloned into AgeI/XhoI digested pEAQ-*HT*. pEAQ-RBD-C538A was generated by cloning of the PCR product obtained with STRINGS-7F/RBD-7R into AgeI/XhoI sites of pEAQ-*HT*. The sequence coding for RBD-215 (amino acids 319-533) was amplified from the synthetic DNA using STRINGS-7F/RBD-9R and cloned into pEAQ-*HT*. To express the RBD-215 mutants RBD-215-1Q, RBD-215-2Q and RBD-215-12Q (Supplementary Figure 1), synthetic RBD DNA fragments harboring the respective codon-exchanges were amplified and cloned in the same manner. For RBD-RFP expression, RBD was amplified from the synthetic RBD DNA fragment with RBD-3F and RBD-5R, XbaI/BamHI digested and cloned into expression vector p48 (Hüttner et al., 2014b).

### Cloning of Expression Vectors for Chaperones and Other Proteins

The pEAQ-CRT expression vector carrying human CRT was described in detail previously (Margolin et al., 2020a). The expression vectors p47-SUBEX-C57Y (SUBEX-C57Y-GFP) (Hüttner et al., 2014a), p31-NbHEXO3 (HEXO3-RFP) (Shin et al., 2017), p59-CRT2 (RFP-CRT2) (Göritzer et al., 2020), p110-CNX1 (RFP-CNX1) (Göritzer et al., 2020), p42-BiP2 (BiP2-HA) (Göritzer et al., 2020) and ST-RFP (Schoberer et al., 2019) were described previously. The *Arabidopsis thaliana* CRT2 (At1g09210) coding sequence was amplified from p59-CRT2 with primers At1g09210-11F/At1g09210-12R and cloned into AgeI/XhoI sites of pEAQ-*HT* to generate pEAQ-AtCRT2 (untagged CRT2). Vector p41-AtCNX1 (CNX1-HA) was generated by PCR amplification from p110-CNX1 with primers CNX1-10F/CNX1-11R and cloning into the XbaI/BamHI sites of p41 (Shin et al., 2018). For p42-CRT3 (CRT3-HA), the coding sequence was amplified from *A. thaliana* cDNA using primers CRT3-3F/CRT3-4R, XbaI/BamHI digested and cloned into XbaI/BamHI digested p42. For p117-PDI5 (RFP-PDI5) the coding sequence was amplified from *A. thaliana* cDNA using primers PDI5-3F/PDI5-4R, XbaI/BamHI digested and cloned into XbaI/BamHI digested p117 (Shin et al., 2018). For p45-AtCDC48A-QQ generation, the coding sequence was PCR amplified with primers AtCDC48-3F/-6R from the AtCDC48A-QQ K487 plasmid provided by Ralph Panstruga (RWTH Aachen University, Aachen, Germany) (Müller et al., 2005). The PCR product was SpeI/SalI digested and cloned into XbaI/SalI sites of vector p45. Expression vector p45 is a derivative of pPT2M (Strasser et al., 2005) with an N-terminal RFP-tag expressed under the control of the CaMV35S promoter.

### Protein Expression and Purification

The pEAQ-*HT* plant expression vectors containing RBD, RBD-KDEL, RBD-C538A, RBD-215, RBD-215-1Q, RBD-215-2Q, and RBD-215-12Q were transformed into *Agrobacterium tumefaciens* strain UIA143 (Strasser et al., 2005). Syringe-mediated agroinfiltration of leaves from 5-week old *N. benthamiana* wild-type or ΔXT/FT was used for transient expression as described (Strasser et al., 2008). For the purification, leaves were harvested 4 days after infiltration and intracellular fluid was collected by low-speed centrifugation as described in detail previously (Castilho et al., 2011). His-tagged RBD or RBD-215 were purified from collected intracellular fluid by loading onto a 5 ml HisTrap HP column (Sigma-Aldrich), elution with imidazole and subsequent dialysis and concentration by ultracentrifugation as described in detail previously (Göritzer et al., 2019). Expression and purification of RBD-His, ACE2-Fc and the monoclonal antibody CR3022 in HEK293 cells has been described in detail recently (Castilho et al., 2021; Klausberger et al., 2021).

### Immunoblot Analysis

For co-expression with different constructs, agrobacteria were mixed, infiltrated into leaves and harvested at the indicated time points. For the block of α-mannosidases, 50 μM kifunensine (Santa Cruz Biotechnology) was co-infiltrated with the agrobacteria suspension and for the block of α-glucosidases 200 μM castanospermine (Sigma-Aldrich) was co-infiltrated. Crude protein extracts or purified protein were subjected to SDS-PAGE under reducing or non-reducing (no reducing agent, no boiling of samples) conditions and after blotting the proteins were detected using anti-His (Thermo Fisher Scientific), anti-RBD (Sino Biological) anti-GFP-HRP (Miltenyi Biotec), anti-RFP (Chromotek) and anti-HA (Roche) antibodies. For deglycosylation, proteins were denatured and incubated with or without Endo H or PNGase F (both from NEB) according to the manufacturer’s instructions.

### Luminex Assay

Receptor binding domain and RBD-215 were separately coupled to MagPlex carboxylated polystyrene microspheres (Luminex Corporation) according to the manufacturer’s instruction, with the following minor modifications: 5 μg of each RBD antigen was used for coupling per one million microspheres. Coupling was performed in a total volume of 500 μL in 96-Well Protein LoBind Deepwell plates (Eppendorf) and plates were incubated at 600 rpm on a Heidolph Titramax 1000 plate shaker (Heidolph). The assays with pre-COVID-19 sera and sera from SARS-CoV-2 infected individuals (AIT cohorts) were carried out as described in detail recently (Klausberger et al., 2021).

### Biolayer Interferometry Measurements

Interaction studies of RBD-215 with in-house produced anti-RBD monoclonal antibody CR3022 (Klausberger et al., 2021) were performed on an Octet RED96e system using high precision streptavidin biosensors (ForteBio). CR3022 was biotinylated using the EZLink Sulfo-NHS-LC Biotin kit (Thermo Fisher Scientific) and purified using PD-10 desalting columns (Cytiva). All assays were conducted in PBS supplemented with 0.05% (v/v) Tween 20 and 0.1% (w/v) BSA (PBST-BSA) at 25°C with the plate shaking at 1000 rpm. The biosensors were equilibrated in PBST-BSA followed by dipping into a 34 nM solution of the respective biotinylated capture molecule. To determine K_*d*_ values, titration of RBD-215 was performed to cover a broad concentration range around the respective K_*d*_ value. To record association rates, CR3022-loaded biosensors were submerged into three-fold (300–1.2 nM) serial dilutions of RBD for 300 s. For dissociation, the biosensors were dipped into PBST-BSA for 100 s. Each experiment was performed in triplicates. Data were evaluated using the Octet data analysis software version 11.1.1.39 as described (Klausberger et al., 2021).

### ELISA

2.5 μg/ml of monoclonal antibody CR3022 (Absolute Antibody) or 3 μg/ml of HEK293-produced human ACE2-Fc (Klausberger et al., 2021) were coated (50 μl/well) in bicarbonate buffer or PBS, respectively, onto NUNC MaxiSorp 96 well plates (Thermo Fisher Scientific) overnight at 4°C. Plates were washed three times with PBS supplemented with 0.1% (v/v) Tween 20 (PBST) and subsequently blocked for 1 h with 1% (w/v) BSA in PBST. Purified RBD was diluted in PBST supplemented with 1% BSA (twofold dilution series: 1–0.0625 μg/ml) and incubated for 2 h. The plates were washed 3 times with PBST and incubated for 1.5 h with biotin-conjugated anti-His antibody (Thermo Fisher Scientific) diluted 1:1000 in PBST + 1% (w/v) BSA. After washing, plates were incubated for 30 min with streptavidin-horseradish peroxidase (HRP) conjugate (Roche) diluted 1:5000 in PBST + 1% (w/v) BSA. After 3 washes, substrate solution [10 mM sodium acetate, pH 5 + 1:60 diluted TMB-stock solution (0.4% (w/v) tetramethylbenzidine (Fluka) in DMSO) + 1:300 diluted H_2_O_2_ (0.6% in H_2_O)] was applied (150 μl/well) and plates were incubated for 5–10 min with shaking. Reactions were stopped by the addition of 1 M sulfuric acid (25 μl/well) and absorbance was measured at 450 nm on a Tecan Sunrise Microplate reader using a reference wavelength of 620 nm.

### Confocal Microscopy

Leaves of 5-week-old wild type *N. benthamiana* were infiltrated with agrobacterium suspensions carrying the plasmids for protein expression with an OD_600_ of 0.1. Confocal images were acquired 72 h after infiltration on a Leica SP5 confocal microscope (Leica Microsystems) as described (Schoberer et al., 2019).

### Liquid Chromatography Electrospray Ionization Mass Spectrometry (LC/ESI-MS)

Purified RBD-215 was S-alkylated with iodoacetamide and digested in solution with endoproteinases LysC (Roche) and GluC (Promega). Digested samples were analyzed using a maXis 4G QTOF mass spectrometer (Bruker) as described (Klausberger et al., 2021).

## Data Availability Statement

The raw data supporting the conclusions of this article will be made available by the authors, without undue reservation.

## Ethics Statement

The studies involving human participants were reviewed and approved by Ethics Committee of the city of Vienna. Written informed consent for participation was not required for this study in accordance with the national legislation and the institutional requirements.

## Author Contributions

Y-JS, JK-B, UV, JS, NFK, MK, EL, CG-G, KV, and MH conducted the experiments. Y-JS, MK, EL, CG-G, AW, ES, LM, and RS analyzed the results. Y-JS, AW, ES, LM, and RS supervised and designed the experiments. EM helped with data analysis and provided expertise for chaperone expression. RS conceptualized the study and wrote the manuscript with support from LM and Y-JS. All authors have made a substantial and intellectual contribution to the work and approved it for publication.

## Conflict of Interest

EM is a named inventor on patent applications describing the use of chaperones to improve protein production in plants. The remaining authors declare that the research was conducted in the absence of any commercial or financial relationships that could be construed as a potential conflict of interest.
