# Supplementary material for: N-Glycosylation of the SARS-CoV-2 Receptor Binding Domain Is Important for Functional Expression in Plants
Source: Front Plant Sci. 2021 Jun 15;12:689104. doi: 10.3389/fpls.2021.689104 (PMC8239413; doi:10.3389/fpls.2021.689104)
Supplement: Supplementary file 1 [file Data_Sheet_1.PDF]

Supplementary Material

N-glycosylation of the SARS-CoV-2 receptor binding domain is important for functional expression in plants

Yun-Ji Shin[1], Julia König-Beihammer[1], Ulrike Vavra[1], Jennifer Schwestka[1], Nikolaus F. Kienzl[1], Miriam Klausberger[2] Elisabeth Laurent[3], Clemens Grünwald-Gruber[4], Klemens Vierlinger[5], Manuela Hofner[5], Emmanuel Margolin[6,7], Andreas Weinhäusel[5], Eva Stöger[1], Lukas Mach[1], Richard Strasser[1,*]

Contents

Supplementary Figure 1. Amino acid sequences of the expressed RBD variants.

Supplementary Figure 2. Immunoblot analysis of RBD-KDEL co-expressed with human CRT or *Arabidopsis* CNX1.

Supplementary Figure 3. Immunoblot analysis of RBD-C538A.

Supplementary Figure 4. SEC elution profile of RBD-215.

Supplementary Figure 5. MS spectra of the two RBD-215 glycopeptides.

Supplementary Figure 6. RBD-215 expression is not affected by co-expression of *Arabidopsis* CRT2 or CNX1.

Supplementary Figure 7. Human CRT does not improve the expression of RBD-12Q lacking both N-glycosylation sites.

Supplementary Figure 8. MS spectra of the two RBD-215 glycopeptides carrying oligomannosidic N-glycans.

Supplementary Table 1. List of primers used in this study.

RBD amino acid sequence

MANKHMSLSLFIVLLGLSCSLASGRVQPTESIVRFP**N**ITNLCPFGEVF**N**ATRFASVYAWNRKRISNCVADYSVL
YNSASFSTFKCYGVSPTKLNDLCFTNVYADSFVIRGDEVRQIAPGQTGKIADYNYKLPDDFTGCVIAWNSNNLD
SKVGGNYNYLYRLFRKSNLKPFERDISTEIYQAGSTPCNGVEGFNCYFPLQSYGFQPTNGVGYQPYRVVVLSFE
LLHAPATVCGPKKSTNLVKNKCVNFHHHHHH

RBD-KDEL amino acid sequence

MANKHMSLSLFIVLLGLSCSLASGRVQPTESIVRFP**N**ITNLCPFGEVF**N**ATRFASVYAWNRKRISNCVADYSVL
YNSASFSTFKCYGVSPTKLNDLCFTNVYADSFVIRGDEVRQIAPGQTGKIADYNYKLPDDFTGCVIAWNSNNLD
SKVGGNYNYLYRLFRKSNLKPFERDISTEIYQAGSTPCNGVEGFNCYFPLQSYGFQPTNGVGYQPYRVVVLSFE
LLHAPATVCGPKKSTNLVKNKCVNFHHHHHHKDEL

RBD-C538A amino acid sequence

MANKHMSLSLFIVLLGLSCSLASGRVQPTESIVRFP**N**ITNLCPFGEVF**N**ATRFASVYAWNRKRISNCVADYSVL
YNSASFSTFKCYGVSPTKLNDLCFTNVYADSFVIRGDEVRQIAPGQTGKIADYNYKLPDDFTGCVIAWNSNNLD
SKVGGNYNYLYRLFRKSNLKPFERDISTEIYQAGSTPCNGVEGFNCYFPLQSYGFQPTNGVGYQPYRVVVLSFE
LLHAPATVCGPKKSTNLVKNKAVNFHHHHHH

RBD-215 amino acid sequence

MANKHMSLSLFIVLLGLSCSLASGRVQPTESIVRFP**N**ITNLCPFGEVF**N**ATRFASVYAWNRKRISNCVADYSVL
YNSASFSTFKCYGVSPTKLNDLCFTNVYADSFVIRGDEVRQIAPGQTGKIADYNYKLPDDFTGCVIAWNSNNLD
SKVGGNYNYLYRLFRKSNLKPFERDISTEIYQAGSTPCNGVEGFNCYFPLQSYGFQPTNGVGYQPYRVVVLSFE
LLHAPATVCGPKKSTNLHHHHHH

RBD-215-1Q amino acid sequence (NIT mutated to QIT)

MANKHMSLSLFIVLLGLSCSLASGRVQPTESIVRFPQITNLCPFGEVF**N**ATRFASVYAWNRKRISNCVADYSVL
YNSASFSTFKCYGVSPTKLNDLCFTNVYADSFVIRGDEVRQIAPGQTGKIADYNYKLPDDFTGCVIAWNSNNLD
SKVGGNYNYLYRLFRKSNLKPFERDISTEIYQAGSTPCNGVEGFNCYFPLQSYGFQPTNGVGYQPYRVVVLSFE
LLHAPATVCGPKKSTNLHHHHHH

RBD-215-2Q amino acid sequence (NAT mutated to QAT)

MANKHMSLSLFIVLLGLSCSLASGRVQPTESIVRFP**N**ITNLCPFGEVFQATRFASVYAWNRKRISNCVADYSVL
YNSASFSTFKCYGVSPTKLNDLCFTNVYADSFVIRGDEVRQIAPGQTGKIADYNYKLPDDFTGCVIAWNSNNLD
SKVGGNYNYLYRLFRKSNLKPFERDISTEIYQAGSTPCNGVEGFNCYFPLQSYGFQPTNGVGYQPYRVVVLSFE
LLHAPATVCGPKKSTNLHHHHHH

RBD-215-12Q amino acid sequence

MANKHMSLSLFIVLLGLSCSLASGRVQPTESIVRFPQITNLCPFGEVFQATRFASVYAWNRKRISNCVADYSVL
YNSASFSTFKCYGVSPTKLNDLCFTNVYADSFVIRGDEVRQIAPGQTGKIADYNYKLPDDFTGCVIAWNSNNLD
SKVGGNYNYLYRLFRKSNLKPFERDISTEIYQAGSTPCNGVEGFNCYFPLQSYGFQPTNGVGYQPYRVVVLSFE
LLHAPATVCGPKKSTNLHHHHHH

Supplementary Figure 1. Amino acid sequences of the expressed RBD variants. The barley α-amylase signal peptide is highlighted in magenta. The SARS-CoV-2 RBD domain (amino acids 319 to 541, RVP…VNF, see UniProt entry P0DTC2) has been selected based on recent publications (Amanat *et al.* 2020; Lan *et al.* 2020). The two N-glycosylation consensus sequences are underlined and the Asn residues (N331 and N343) are shown in bold. Cysteine residues are shown in red, the 6x histidine-tag is highlighted in yellow and the KDEL Golgi-to-ER retrieval signal is highlighted in green. In the RBD-215 mutants, Asn residues were exchanged to Gln (highlighted in red) and in RBD-C538A the exchanged amino acid is highlighted in light blue.

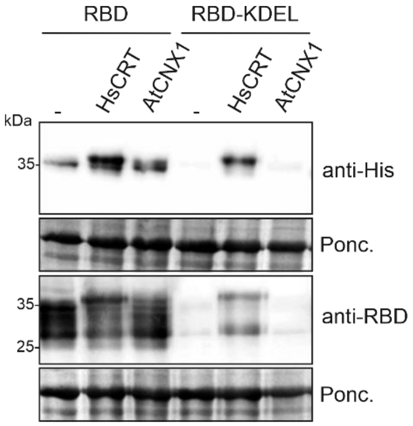

Supplementary Figure 2. Immunoblot analysis of RBD-KDEL co-expressed with human CRT or *Arabidopsis* CNX1. RBD-KDEL was transiently co-expressed in *N. benthamiana* with human CRT (HsCRT) or *Arabidopsis* CNX1 (AtCNX1). Proteins were extracted 4 days after infiltration and subjected to SDS-PAGE and immunoblotting with the indicated antibodies. RBD expression was included as a control. Ponceau S staining (Ponc.) is shown as a loading control.

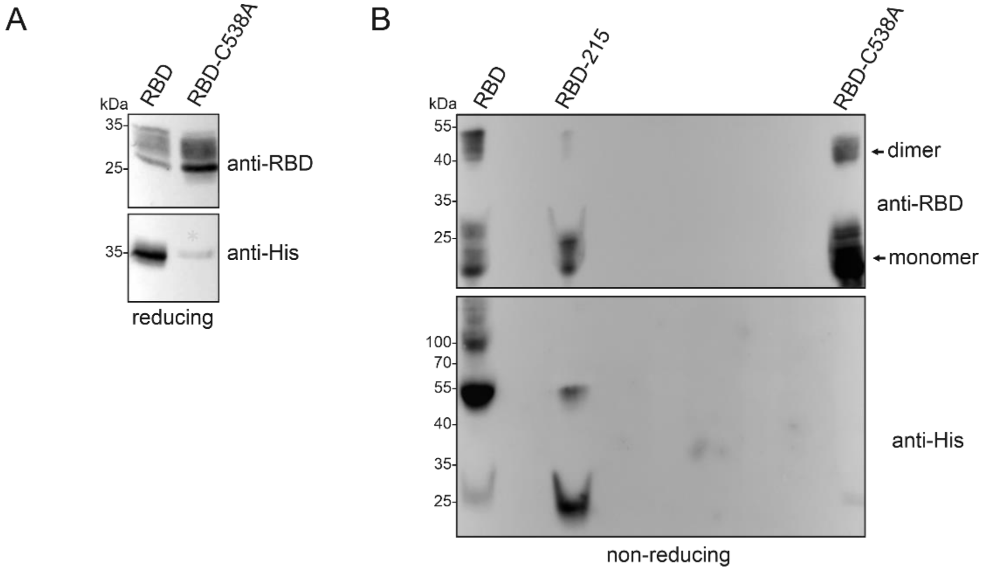

Supplementary Figure 3. Immunoblot analysis of RBD-C538A. RBD-C538A was transiently expressed in *N. benthamiana*. Proteins were extracted 4 days after infiltration and subjected to SDS-PAGE under (A) reducing and (B) non-reducing conditions. Expression of RBD and RBD-215 was included as controls.

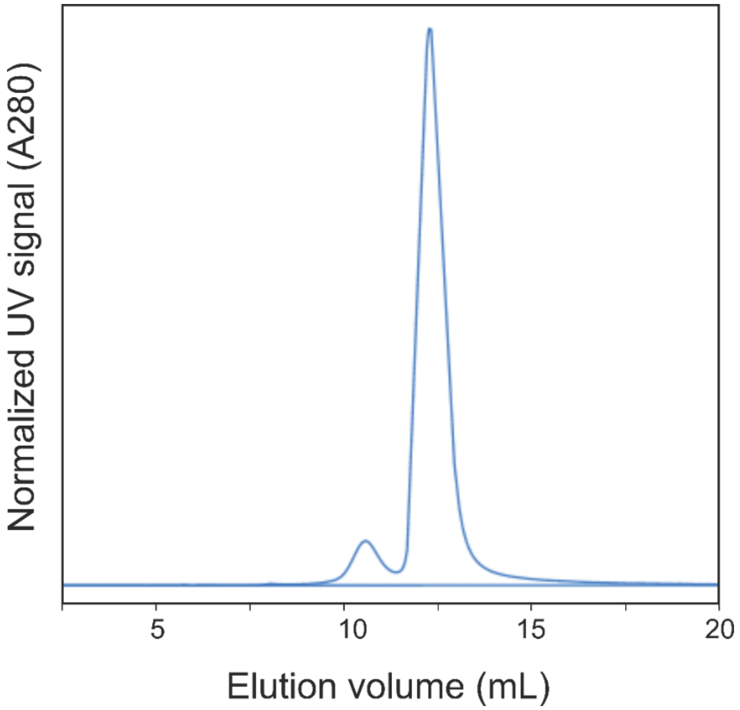

Supplementary Figure 4. SEC elution profile of RBD-215. Recombinant RBD-215 was IMAC purified from the apoplast of infiltrated *N. benthamiana* and subjected to SEC. The smaller peak represents the dimeric form.

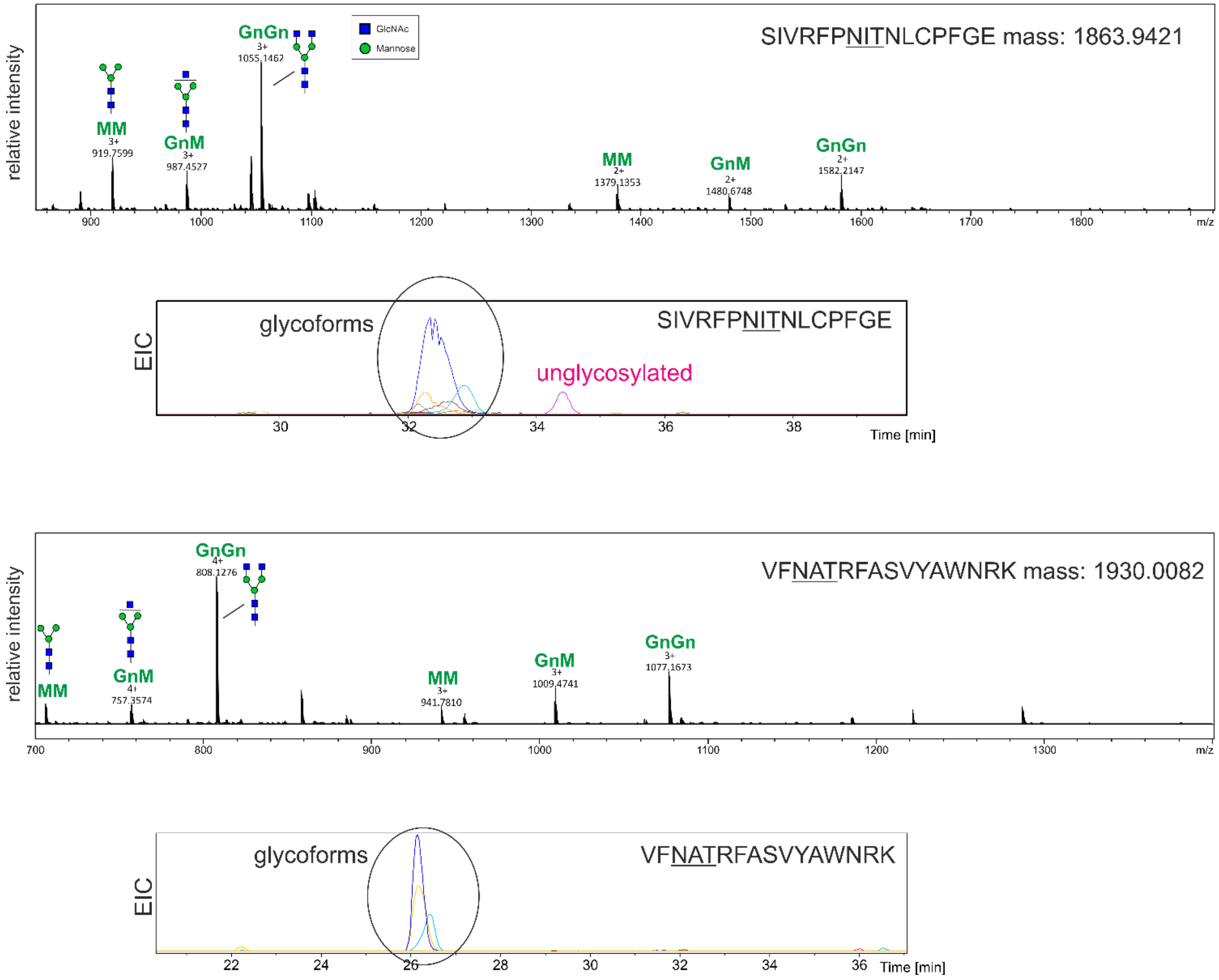

Supplementary Figure 5. MS spectra of the two RBD-215 glycopeptides. RBD-215 was purified by IMAC from the apoplastic fluid 4 days after infiltration. Purified RBD-215 was LysC and GluC digested and analysed by MS. The glycopeptides SIVRFPNITNLCPFGE (carrying N331) and VFNATRFASVYAWNRK (carrying N343) are shown. The assigned N-glycans are illustrated with cartoon presentations. The extracted ion chromatograms (EIC) of the most abundant glycopeptides and the non-glycosylated peptides carrying N-glycosylation sites N331 and N343 are shown.

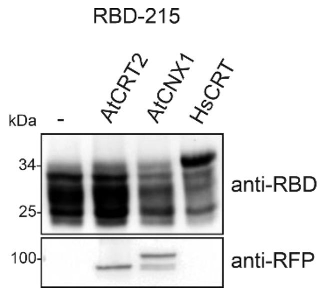

Supplementary Figure 6. RBD-215 expression is not affected by co-expression of *Arabidopsis* CRT2 or CNX1. (A) RBD-215 was co-expressed with *Arabidopsis* CRT2 (fused to RFP, AtCRT2), *Arabidopsis* CNX1 (fused to RFP, AtCNX1) or HsCRT.

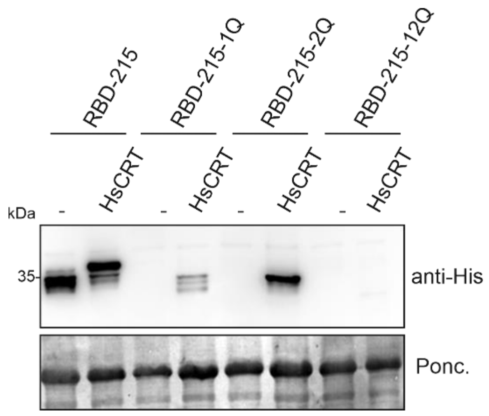

Supplementary Figure 7. Human CRT does not improve the expression of RBD-215-12Q lacking both N-glycosylation sites. RBD-215-12Q was expressed in the presence or absence of human CRT (HsCRT). Proteins were extracted 4 days after infiltration and subjected to immunoblotting. RBD-215, RBD-215-1Q and RBD-215-2Q were included as controls. Ponceau S staining (Ponc.) is shown as a loading control.

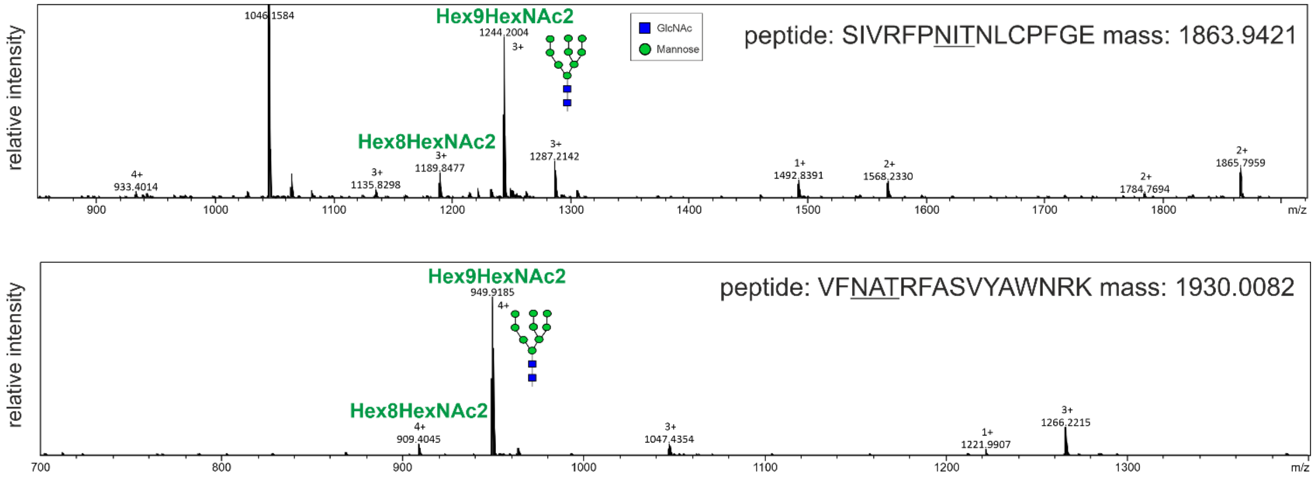

Supplementary Figure 8. MS spectra of the two RBD-215 glycopeptides carrying oligomannosidic N-glycans. RBD-215 was co-infiltrated with kifunensine and purified 3 days after infiltration from the apoplastic fluid. Purified RBD-215 was LysC and GluC digested and analysed by MS. The glycopeptides SIVRFPNITNLCPFGE (carrying N331) and VFNATRFASVYAWNRK (carrying N343) are shown. In each spectrum, the most prominent N-glycan peak is illustrated with a cartoon presentation.

Supplementary Table 1. List of primers used in this study.

Name	Sequence (5' – 3')
STRINGS-7F	CTTCCGGCTCGTTTGACCGGTATG
STRINGS-8R	AAAAACCCTGGCGCTCGAG
RBD-3F	TATATCTAGAATGGCCAACAAGCACATGAG
RBD-5R	TATAGGATCCGAAGTTCACGCACTTGTTCTT
RBD-6R	TATACTCGAGCTACAGTTCGTCCTTGTGGTGGTGATGGTGATGGA
RBD-7R	TATACTCGAGCTAGTGGTGGTGATGGTGATGGAAGTTCACGGCCTTGT TCTTGAC
RBD-9R	TATACTCGAGCTAGTGGTGGTGATGGTGATGACCCAGGTTGGTAGACT TCTTAGGA
At1g09210-11F	TATAACCGGTATGGCGAAAATGATTCCTAGCC
At1g09210-12R	TATACTCGAGCTATAGCTCATCATGAGCGGTGGCG
CNX1-10F	TATATCTAGAGACGATCAAACGGTTCTGTATG
CNX1-11R	TATAGGATCCCTAATTATCACGTCTCGGTTGCC
PDI5-3F	TATATCTAGAGAAGAGACGGAGACGAAGGAG
PDI5-4R	TATAGGATCCTCAGAGCTCATCCTTGACTTCC
AtCDC48-3F	TATAACTAGTTCTACCCCAGCTGAATCTTCAGAC
AtCDC48-6R	TTATAGTCGACCTAATTGTAGAGATCATCATCGTC
CRT3-3F	TATATCTAGAATGGGATTACCTCAAAATAAGCTC
CRT3-4R	TATAGGATCCGTAATCATCCATATAATCACGTGGA
